# A review of Kenya’s cancer policies to improve access to cancer testing and treatment in the country

**DOI:** 10.1186/s12961-019-0506-2

**Published:** 2020-01-07

**Authors:** Louise Kathini Makau-Barasa, Sandra Greene, N. A. Othieno-Abinya, Stephanie B. Wheeler, Asheley Skinner, Antonia V. Bennett

**Affiliations:** 1 Department of Health Policy and Management, Gillings School of Global Public Health, University of North Carolina, Chapel Hill, NC United States of America; 2 University of Nairobi, Nairobi, Kenya; 3 Duke University, Durham, NC United States of America

**Keywords:** Cancer policy, Health systems, Health services access, Cancer, Testing, Treatment, Low-resource setting, Kenya, Africa

## Abstract

**Background:**

Cancer is the third-leading cause of mortality in Kenya, resulting in unique challenges to the country’s health system. An increase in the number of cancer cases in Kenya over the past decade resulted in legislative actions and policies to guide delivery of cancer services. Kenya’s new national cancer control strategy and past policy efforts provide an opportunity to synergise information and enhance understanding to improve cancer diagnosis and treatment in the country. The objectives of this study are to (1) document policy-modifiable factors based on a review of policy documents and results of a key informant survey and (2) develop recommendations to improve policies affecting cancer testing and treatment services in Kenya. This study builds upon our previous study Improving Access to Cancer Testing and Treatment in Kenya (Makau Barasa et al. *J Global Oncol* 2(216), 2017).

**Methods:**

The study applied an in-depth systematic review of Kenya’s cancer policies and guidelines, a qualitative analysis of results from a section of a semi-structured key informant survey focused on the opinions of clinicians delivering cancer services as well as cancer support groups and advocacy leaders, and a stakeholder analysis identifying key policy-makers and implementers. Details of the complete key informant survey were published in our previous study.

**Results:**

Kenya’s cancer policies have guided progress made in providing the legal and implementation frameworks for the development and delivery of cancer services at the national and county levels. Some policy implementation gaps are noted. These include inadequate financing for cancer services, limited research and data to support policy formulation, and the concentration of cancer services in urban areas. The key informant survey identified policy-modifiable actions that can address some of the gaps and improve the delivery of and access to cancer testing and treatment services in the country. Some of these include addressing the financial barriers affecting cancer testing and treatment services; increasing stakeholder engagement in training health personnel to deliver cancer testing and treatment services; decentralising cancer services and improving cancer surveillance and research; and increasing education and awareness about cancer symptoms, screening procedures and treatment options. A set of priority policy actions were selected from the study findings and used to develop recommendations for Kenya’s policy-makers and stakeholders.

**Conclusions:**

Revisions to Kenya’s cancer policies are seeking to address gaps noted in past policies and to improve access to cancer testing and treatment in Kenya. However, based on study findings, additional actions can be taken to strengthen policy implementation. Considering the policy formulation and implementation process and costs, this study recommends focusing on three priority policy actions that can have significant impact on improving access to cancer testing and treatment services. These include addressing financing, insurance and human resources gaps; increasing stakeholder engagement; and decentralising health services for better surveillance and data to inform policies.

## Introduction

According to WHO, cancer is the third-leading cause of mortality in Kenya after infectious and cardiovascular diseases [1]. In 2011, Kenya’s National Cancer Control Study reported an estimated 37,000 new cancer cases and 28,500 cancer deaths in the country. In 2018, the International Agency for Research on Cancer Global Cancer Incidence, Mortality and Prevalence (GLOBOCAN) data projected an increase to 47,000 new cancer cases and 32,987 cancer deaths in Kenya.

The growth of cancer cases in Kenya over the past decade has resulted in legislative actions and policies to guide the delivery of cancer testing and treatment services, including the development of the country’s first strategic plan for cancer — Kenya National Cancer Control Strategy: 2011–2016 — by the Ministry of Public Health and Sanitation and the Ministry of Medical Services in 2011. These guidelines identified available national resources and infrastructure while advocating for increased investments to improve cancer services. These efforts were followed by the establishment of a legal framework to support cancer services through legislative action — the Cancer Prevention and Control Act, 2012. These efforts laid the framework for the establishment of the National Cancer Registry and the National Cancer Institute – Kenya (NCI-K). In 2015, the Cancer Act, 2012, was amended to include County Cancer Prevention and Control Committees and renamed the Cancer Prevention and Control (Amendment) Act, 2015.

In 2013, Kenya’s Ministry of Health (MoH) issued the National Guidelines for Cancer Management Kenya, highlighting the required cancer treatment procedures, standards and qualities to be applied by Kenya’s healthcare practitioners. In 2014, the MoH, with support from the United States National Cancer Institute (NCI-US) Center for Global Health, convened the Kenya Cancer Research and Control Stakeholder Meeting. The objective was to provide guidance on setting priorities for Kenya’s cancer sector and invite participants to the national cancer policy revision process [2–4]. This and other stakeholder efforts resulted in the development of Kenya’s National Cancer Control Strategy (NCCS): 2017–2022 in 2017. Other policy documents also serve as auxiliary guidelines on the diagnosis, prevention, control and treatment of specific cancers in the country (Table 1).

**Table 1:** A list of Kenya’s cancer policies and guidelines

Document	Objective
1. The Cancer Prevention and Control Act, 2012 (No. 15 of 2012). The National Council for Law Reporting with the Authority of the Attorney-General. 2012	This document lays out the legal framework for the development of cancer prevention, treatment and control interventions, and to define the role of the national and county governments in delivering these services
2. The Cancer Prevention and Control (Amendment) Bill, 2015: An amendment to the Cancer Act (2012)	This bill seeks to clarify roles and responsibilities of the National Cancer Institute and the county-level functions in the delivery of cancer services
3. National Cancer Control Strategy. 2011–2016 -Government of Kenya Ministry of Public Health and Sanitation and Ministry of Medical Services [5].	This document outlines interventions to be undertaken by the government and other partners to enhance existing structures and pull together additional resources to address cancer in Kenya
4. National Guidelines for Cancer Management Kenya [6]	A detailed guideline for clinicians delivering cancer screening, diagnostic and treatment services for more than 20 types of cancer in Kenya; this guideline provides cancer staging guidelines, treatment modalities and lists potential drugs that can be administered for the cancer
5. National Guidelines for the Prevention and Management of Cervical, Breast and Prostate Cancer [7]	Treatment and palliative guidelines for clinicians delivering cancer services – screening, diagnosis and management of cervical, breast and prostate cancers in Kenya
6. National Cervical Cancer Prevention Program: Strategic Plan 2012–2015	A strategic framework and priority actions for cervical cancer prevention with the aim of reducing the incidence of cervical cancer in Kenya
7. National Palliative Care Guidelines, 2013, Ministry of Health	The guideline identifies key areas and suggests interventions to promote provision of holistic quality palliative care in Kenya
8. The Kenya National Patients’ Rights Charter, 2013, Ministry of Health	Guidelines to inform patients about their rights and responsibilities when seeking quality healthcare services; also serves as a conflict resolution guideline for patients and healthcare providers
9. The National Cancer Control Strategy 2017–2022 [8]	This document sets objectives to reduce Kenya’s cancer burden, considers the decentralisation of health services and new cancer treatment financing options

### Study aims and importance

The aims of this study are to (1) document policy-modifiable factors based on a review of policy documents and results of a key informant survey and (2) develop recommendations to improve policies affecting cancer testing and treatment services in Kenya based on results from the policy review, key informant survey and a stakeholder analysis.

Increased national focus on cancer control, prevention and treatment as well as the release of Kenya’s new national cancer control strategy provide an opportunity to synergise information into a comprehensive understanding of how to improve cancer diagnosis and treatment in the country. This study contributes to literature on improving cancer services in sub-Sahara African countries with similar economic and demographic challenges affecting their delivery of cancer services.

## Materials and methods

A retrospective analysis of Kenya’s cancer policies was conducted with the aim of developing a contextual understanding of Kenya’s policy development and implementation environment. The analysis was informed by methodological approaches to conducting health policy analysis by Walt et al. [9]. The analysis forms part of our previous study focused on identifying barriers faced by patients seeking cancer testing and treatment services, and clinicians delivering the services in Kenya [10].

Data for this study was collected through (1) a systematic review of Kenya’s cancer policies and guidelines; (2) an analysis a key informant survey focused on the policy opinions of seven clinical oncologists and seven cancer patient support and advocacy group leaders in Kenya; and (3) a stakeholder analysis that identified key groups in Kenya’s policy development and implementation process and also informed by research and consultations during the proposal development phase of this study in 2014–2015, information obtained from the Kenya Cancer Research and Control National Stakeholder Meeting Brief held in May 2014 in Kenya, and Guidelines for Conducting a Stakeholder Analysis [11]. The analysis included understanding their roles, levels of influence and interest in the policy development and implementation process with the aim of proposing a communication and engagement strategy that would enhance each stakeholder group’s support of the proposed policy actions.

The key informant survey was conducted from December 2015 to January 2016. The survey was designed to respond to the three research questions, shown below, that formed our larger study on barriers to cancer testing and treatment in Kenya.

1. What are the patient characteristics, including level of cancer awareness, among people seeking access to cancer testing and treatment services, and which of these are modifiable through policy or other actions?
2. How does the organisation of health services and the health infrastructure affect access for patients?
3. What policy actions can improve access to timely cancer testing and treatment in Kenya?

The Andersen and Aday Conceptual Framework on Health Access [12] was utilised to analyse data from the survey and to respond to the key study question — ‘How can access to cancer diagnosis and treatment be improved in Kenya?’ and the sub-question focused on policy ‘What policy actions can improve access to timely cancer testing and treatment in Kenya?’. The framework was used to triangulate findings from the policy analysis, survey and stakeholder analysis, and inform the policy recommendations. A detailed description of the framework and its application was presented in our abovementioned study.

## Results

### Key informant survey findings

This section presents survey findings in response to research question 3 — ‘What policy actions can improve access to timely cancer testing and treatment in Kenya?’ A total of 45 potential study participants were identified and informed about the study in 2015. These were contacted and 14 were qualified based on the eligibility criteria of being a clinician or leader of a cancer support and advocacy group. Table 2 presents a summary of the 14 study participants’ characteristics.

**Table 2:** Characteristics of the study participants

	Clinicians	Cancer support and advocacy group leaders
Total *n* = 14	7	7
Male	3	3
Female	4	4
Type of organisation		
Public hospital	2	–
Private hospital	4	–
Mission hospital	1	–
Non-government organisation	0	7

Structural coding, based on a qualitative data analysis approach by Patel [13], was used to identify the following seven main barriers to patients seeking cancer testing and treatment regardless of the type of cancer: (1) high cost of testing and treatment partly due to insurance limitations; (2) the population’s and clinicians’ low level of knowledge about cancer; (3) the population’s poor health-seeking behaviours; (4) long distances to access cancer services; (5) lack of decentralised diagnosis and treatment facilities; (6) poor provider-to-patient communication; and (7) the need for better cancer policy development and implementation. Interview methods and results from this survey were published in our previous study.

A limited number of policy recommendations were formulated based on these study findings. These included:

1. Focusing on the financing, insurance and developing human resources
2. Increasing stakeholder engagement
3. Decentralising services and improving cancer surveillance and data from the county level

However, some of these recommendations are currently being implemented as part of the NCCS 2017–2022.

### Policy literature review findings

Kenya’s cancer policy and guideline documents were identified through an internet search and consultations with Kenya’s MoH. These documents lay the foundation for the implementation of all other cancer guidelines noted in Table 1 and include two main policy documents — the National Cancer Control Strategy 2017–2022 and the National Guidelines for Cancer Management in Kenya [6]. These two documents provide the implementation framework for the development and delivery of cancer services, including testing and treatment. A third key document, the Cancer Act, 2012 (amended 2015), provides the legal framework for cancer services and defines the roles of national and county governments in the delivery of cancer services. These three key documents were reviewed for feasibility and potential effectiveness within Kenya’s current health sector and infrastructure and also due to their role in supporting the cancer policies and guidelines listed in Table 1.

### The National Cancer Control Strategy

In 2017, a revised NCCS 2017–2022 was published by the MoH. This revised NCCS addresses some of the gaps in the 2011–2016 NCCS, including increased focus on the decentralisation of cancer services. Key features of this policy include attention to financing the development and delivery of Kenya’s cancer sector, prioritisation of cancer surveillance, cancer research and personnel training, and availing affordable cancer treatment drugs to patients. It also seeks to align proposed interventions with local, regional and international policies, namely the Kenya Health Policy 2014–2030, the 2011 Brazzaville Declaration on Non-Communicable Diseases Prevention and Control in the WHO African Region, and the WHO Global Action Plan for Prevention and Control of Non-communicable Diseases 2013–2020 [14].

The policy, targeting government and non-governmental agencies delivering cancer services, prioritises a set of evidence-based interventions to improve cancer prevention and control in the country. It outlines five pillars (focus areas) through which the development of cancer services, infrastructure and personnel, and stakeholders’ roles and responsibilities are noted. These are (1) Prevention, Early Detection and Cancer Screening, which draws attention to the importance of primary prevention and early detection of cancers in the country; (2) Cancer Diagnosis, Registration and Surveillance, which emphasises the need for timely diagnosis that can lead to better health outcomes for cancer patients; (3) Cancer Treatment, Palliative Care and Survivorship, which draws attention to the need for effective treatments and palliative care while also addressing cultural factors surrounding end-of-life care; (4) Coordination, Partnership and Financing for Cancer Control, which focuses on improving the coordination of cancer services in the country’s health system, advocates for free cancer care for children below the age of 12 years, and calls for strengthening the NCI-K and the National Cancer Control Program; and (5) Monitoring, Evaluation and Research. which focuses on strengthening the country’s cancer research capacity and evaluating activities to be undertaken under the current strategy. This policy fails to articulate actions to reduce cancer testing and treatment costs, which remain the biggest barriers to delivering cancer services in the country. This also includes measures to reduce the cost of cancer drugs.

An implementation timeline for some of the activities initially identified by this study is noted in Kenya’s NCCS 2017–2022. However, the timeline fails to address critical issues such as expanding human resource capacity for screening and early diagnosis. As a central issue in the successful and sustainable delivery of cancer services in the country, the government and its stakeholders need to commit resources to address the lack of enough personnel in the country’s cancer sector.

However, unlike the previous strategy, this strategy notes plans to develop information, education and communication material to address myths and misconceptions about cancer. The plan also includes efforts to improve the two main tertiary cancer referral centres — Kenyatta National Hospital, Nairobi, and Moi Teaching and Referral Hospital (MTRH), Eldoret — through infrastructure and equipment upgrades as well as the establishment of four comprehensive regional cancer treatment centres in Mombasa, Nakuru, Nyeri and Kisii counties. The implementation framework outlining interventions, timelines and the roles of various stakeholders helps highlight key sector players and the need for resources.

### The National Guidelines for Cancer Management

The National Guidelines for Cancer Management were developed in 2013 by Kenya’s MoH. These guidelines consolidated previous policies and guidelines issued by the Ministry of Public Health and Sanitation and Ministry of Medical Services in 2012, namely the National Guidelines for the Prevention and Management of Cervical, Breast and Prostate Cancer; the National Cervical Cancer Prevention Program: Strategic Plan 2012–2015; the National Clinical Management and Referral Guidelines Volume III [15]; the Kenya Health Policy, 2012–2030; and the NCCS 2011–2016. These revised guidelines target clinicians and contain site-specific approaches on the epidemiology, diagnosis, staging, treatment and prognosis of most adult and paediatric cancers.

By targeting clinicians, in a country with insufficient healthcare personnel, these guidelines fail to recognise the roles of other healthcare workers involved in supporting cancer testing and treatment-related procedures. These include cancer screening as part of improving access to testing and home-based palliative care for some of the patients under treatment. However, this document, together with other guidelines noted in Table 1, has enabled further development of detailed diagnosis and treatment guidelines for prostate, breast and cervical cancers as the most prevalent cancers in the country.

### The Cancer Act, 2012 (amended 2015)

Established through an act of parliament in 2012, the Cancer Act sets the legal framework for the establishment of the NCI-K to oversee and coordinate cancer treatment and diagnostic services in the country [16]. In 2015, the 2012 Cancer Act was amended to provide legislative authority to county councils to formulate and implement county-specific policies on cancer prevention and control under Kenya’s devolved governance. The 2015 amendments also included new provisions aimed at protecting consumers (patients) against discriminatory practices (part IV, Discriminatory Practices) and to develop consumer information and education about cancer (part V – Education and Information). The Act is designed to also promote cancer surveillance by collecting and reporting on cancer cases through referral hospitals and ensuring that counties have the capacity to implement national cancer policies, including equipping facilities to diagnose and treat cancer, train health personnel, educate the population about cancer, and take measures to prevent cancer.

However, the Cancer Act supports the establishment of county cancer centres without noting the need for due diligence to justify their implementation, which risks resulting in the inefficient use of resources and creating cancer centres without adequately skilled personnel and infrastructure in regions with low levels of cancer cases. Overall, the Cancer Act resulted in the formal recognition of cancer as a public health issue meriting legislative action and government support. The Cancer Act also set the legal framework for the management and financing of Kenya’s cancer and mandated the NCI-K to oversee and coordinate cancer treatment and diagnostic services at the national level.

A review of these three key policy documents indicates actions taken by Kenya’s government and stakeholders in response to the country’s increased demand for cancer testing and treatment services.

### Stakeholder analysis

The stakeholder analysis identified groups that yield considerable influence on Kenya’s cancer policy formulation and implementation, namely (1) national and county governments (public sector); (2) non-governmental organisations (civil society); (3) private sector (pharmaceutical and insurance companies); (4) academia and research groups; (5) media; and (6) international groups. Table 3 presents a summary of these stakeholders’ roles and levels of influence.

**Table 3:** Summary of Kenya’s cancer sector stakeholders, their roles and levels of influence

Stakeholder	National and county government (public sector)	Non-governmental organisations (civil society)	Private sector	Academic and research institutions	Media	International groups
Entity	GOK through the MoH, NCI county-level cancer prevention and control groups	Examples include KENCO, Women4Cancer, Hope Beyond Cancer, Childhood Cancer Initiative, and others	Pharmaceutical and insurance companies	Kenya Medical Research Institute, AMREF, University Hospitals in Kenya	Local TV stations Kenyan newspapers Kenyan Public health journals	WHO/International Agency on Research on Cancer American Cancer Society AMPATH
Role	Creates policies, rules and regulations, enforces implementation, allocates resources to implement policies	Inform policy through patient advocacy, facilitate access to information for cancer patients, raise public awareness about cancer, prevention and treatment options	Influence policy, rules and regulations, and cancer service delivery	Communicate to and on behalf of the public and policy-makers, raise awareness about the availability of cancer diagnostic and treatment services Inform policy through research, implementation evaluation to promote evidence-based policies	Inform and educate the public about cancer, national policies and mobilise groups for action	Inform policy through financial and technical resources and research
Level of Influence	High, GOK and MoH have the power to authorise, implement, finance or reject policies	High, Representing civil society, these groups engage with policy-makers, provide compelling information on cancer to policy-makers and the public, mobilise communities for cancer screening activities, can disseminate information on treatment locations	High Kenya’s private health and public health services sector’s power to influence implementation in the public and private health facilities	Medium low The cancer research is still developing; research findings need to inform policy development and implementation to a larger extent	Medium high As the main source of information for the public, the media can strongly influence the public’s knowledge of cancer and health-seeking behaviour	Medium Recent technical and financial support by the American Cancer Society and the consortium of universities through AMPATH at MTRH has led to increased screening and treatment for a subset of the patient population

*AMPATH* Academic Model Providing Access to Healthcare, *GOK* Government of Kenya, *KENCO* Kenya Network of Cancer Organizations, *MoH* Ministry of Health, *MTRH* Moi Teaching and Referral Hospital, *NCI* National Cancer Institute

The government, represented by NCI-K and the county governments, yields the highest level of influence due to their role in developing, approving and allocating human and financial resources for the implementation of national policies.

Non-governmental organisations (civil society), including Kenya Network of Cancer Organizations (KENCO) and patient support and advocacy groups, have been instrumental in demanding and informing changes to Kenya’s cancer policies. Representing consumers (and patients) they have exerted pressure on legislative actions to include insurance coverage for cancer patients and advocated for lowering the costs of treatment and cancer drugs in the country.

The private sector comprising of health insurance companies, private health facilities and pharmaceutical companies yield significant power in influencing the implementation of Kenya’s policies designed to ensure access to cancer services. Their influence is exerted on informing both private and public health insurance coverage for cancer patients. These entities also inform the formulation of reimbursement rates for all cancer diagnosis and treatment procedures, drug coverage costs and co-pays within the country and for Kenyan patients seeking treatment overseas.

Academia and research institutions inform policy formulation based on studies of the outcomes and impact of some of the policy actions. These include Kenya Medical Research Institute (KEMRI) and Kenya’s university hospitals. In this role, they inform legislature on policy actions that affect patients and clinicians delivering cancer services.

Media comprising of news outlets and social media sites disseminate cancer information to the public and policy-makers. In recent years, Kenya’s media has highlighted inadequacies in the delivery of cancer services in the country, raised public awareness about cancer treatment costs and increased pressure on the government to address challenges faced by patients seeking cancer testing and treatment in the country.

International groups, such as the American Cancer Society, the World Bank and Clinton Health Access Initiative, have been instrumental in providing financial and technical resources in the development of some of Kenya’s cancer policies, cancer registries and human resources. The United States government, through six of its NCI-Designated Cancer Centers, has partnered with Kenyan academic and research organisations to increase cancer research. Participating institutions include the Academic Model Providing Access to Healthcare (AMPATH) based in MTRH, University of Nairobi (UoN) as part of Kenyatta National Hospital, KENCO and KEMRI.

Civil groups represented by community and faith-based organisations, including patient support and advocacy groups, play a significant role in advocating for better access to cancer services and the implementation of policies that are favourable to cancer patients. In addition, these groups conduct public education campaigns and, in some cases, screenings for cervical and breast cancer in collaboration with community health facilities.

Altogether, these stakeholders exert varied levels of influence on the formulation and implementation of Kenya’s cancer policies (Table 3). Understanding their roles and levels of influence is critical in determining the viability of recommendations to improve the current policies.

## Discussion and recommendations

A review of Kenya’s main cancer policies and guidelines, results of the key informant survey and stakeholder analysis identified policy-responsive actions that could improve access to cancer testing and treatment in the country if implemented. Current government actions, such as allocating financial and human resources to the NCI-K and the National Cancer Control Program, indicate government goodwill to improve the delivery of cancer services in the country. The establishment of County Cancer Coordinating Committees recognises the need to improve access and decentralise cancer services for rural populations. However, costs remain a key factor in the acquisition of equipment, health personnel training, cancer treatment and palliative care drugs, and improving infrastructure at national and county-level health facilities delivering cancer services.

Prioritising policy actions is important in allocating limited resources and scaling implementation enables stakeholders to learn, improve and build momentum. This is important in demonstrating a record of accomplishment and successful implementation when seeking additional support within and outside the country. Consequently, a limited number of recommended priority actions to improve access to cancer testing and treatment in Kenya, based on study findings, are presented below.

### Financing, insurance and developing human resources

Kenya’s healthcare financing approach needs to be reviewed and revised to respond to the needs of cancer patients and to establish a health system capable of providing timely and quality cancer care to its population. This includes the abolition of discriminatory practices in the form of coverage limits and bureaucracies by the National Health Insurance Fund (NHIF) and private insurance firms that result in delayed diagnosis, incomplete cancer treatments and inadequate follow-ups that contribute to poor outcomes for cancer patients.

A review of Kenya’s economy and a comparison with neighbouring countries indicate its ability to increase funding allocated to cancer services. According to the World Bank 2017 data, Kenya had the highest GDP in East Africa, valued at US$63,398 million. Kenya spends 3.5% of its GDP on health, with a smaller portion allocated to cancer services, compared to Tanzania (5.8%), Uganda (7.2%) and Rwanda (7.5%). The country also has the lowest health expenditure per capita (US$77) in the region [1].

With 36% of the population living below the international poverty line of US$1.90 per day in 2011, purchasing power parity [17, 18], access to affordable cancer testing and treatment remain out of reach for the majority of the population. While the country has made strides following the inclusion of cancer patients in NHIF and private insurance schemes, limits on the number of treatments, based on the type of cancer, modality and costs of drugs, often result in incomplete treatments for patients [19] and contribute to poor outcomes.

Insufficient financing of cancer services, health system infrastructure and human resource development is one of the main problems faced by low- and middle-income countries like Kenya seeking to implement policies to improve the delivery of cancer services.

External funding and partnerships can accelerate the development and delivery of timely cancer services in the country. Recent initiatives include financial and technical support from the NCI-US during the 2016–2017 fiscal year that was applied to strengthen cancer surveillance and diagnostics in the country following priorities set at the 2014 MoH-hosted Cancer Research and Control Stakeholder Engagement Meeting, and a grant from the World Bank and the Clinton Health Access Initiative that was used to fund the training of Kenyan pathologists through the UoN and the Aga Khan Foundation in 2016–2017. In 2017, Merck Pharmaceuticals, in partnership with Tata Memorial Hospital, India, established Africa’s first medical oncology fellowship program at UoN. Through this initiative, oncology nurses will be trained at UoN and several oncology doctors will receive training at the Tata Memorial Hospital in India.

Health personnel training, accompanied by the simultaneous acquisition of cancer diagnosis and treatment equipment, can accelerate the development of skilled personnel to deliver timely cancer diagnosis and treatment in the country.

### Increasing stakeholder engagement

Kenya’s government has intensified its engagement with stakeholders, including civil society groups, the media, academia and research entities, and international partners. This engagement has resulted in an improved strategic plan (NCCS 2017–2022), which addresses some of the gaps of past policies that included the lack of county-level health ministries in implementing national cancer policies. Actions to strengthen stakeholder engagements can include encouraging policy-makers to engage with civil society groups in cancer policy formulation, implementation and evaluation.

KENCO’s past efforts contributed to changes in NHIF to include cancer patients in coverage schemes beginning late 2015. However, gaps still exist based on the current public and private insurance schemes that limit cancer treatment sessions, resulting in partial cancer treatments with poor outcomes for patients. KENCO and its members are strategically positioned to influence legislative actions that have the potential to address these issues and improve the delivery of cancer testing and treatment services in the country.

### Engaging with the media and civil society groups to educate the public about cancer

The high levels of stigma associated with cancer affect efforts to mobilise the public and demand the government for better cancer testing and treatment services. However, as one of the main sources of information about cancer in the country, the media and civil society groups are also well positioned to lead public education about cancer prevention as well as testing and treatment procedures. This can result in better patient–clinician interactions and promote informed engagement with policy-makers as well as demand for accessible and affordable cancer services from the government.

### Support and encourage research to inform policy actions and outcomes

KEMRI, MTRH and other research entities in the country have data on the incidence and prevalence of cancer in the country based on a limited number of hospital-based cancer registries and the Nairobi Cancer Registry. However, the country lacks a national cancer registry to consolidate data on general and specific cancer rates and trends. This data gap poses challenges to the formulation of effective policies and the allocation of policy implementation resources. Efforts to establish a national cancer registry are noted in the NCCS: 2017–2022; however, financial constraints have delayed its creation. As a short-term measure, the government can engage with research entities in the country and use currently available data to formulate evidence-based policies and priorities that respond to the population’s cancer testing and treatment needs.

### Encourage collaboration with stakeholders to increase cancer testing and treatment capacity

The government of Kenya has cited limited financial and technical capacity to implement some of the policy actions that could improve access to cancer testing and treatment services in the country. These include training oncologists and other clinical personnel required to diagnose and treat cancer. In response, stakeholders such as the UoN, MTRH and pharmaceutical companies are providing technical training and resources to develop clinical personnel for cancer services; these include the American Cancer Society, AMPATH and Merck pharmaceutical’s Medical Oncology Fellowship. Altogether, these are critical in training health personnel to deliver cancer services and achieve the policy goals outlined in the NCCS 2017–2022. Establishing meaningful partnerships with additional stakeholders could accelerate the implementation of cancer policies and improve access to cancer services in the country.

### Decentralising services and improving cancer surveillance and data

The Cancer Act, 2012 (amended 2015), provides the legislative framework for the decentralisation of health services, including cancer testing and treatment in the country. Together with the NCCS 2017–2022, these policies address current gaps seen in the concentration of cancer services in Nairobi and major towns (Mombasa, Kisumu and Eldoret) and the need for a national cancer registry. However, the establishment of county-level cancer diagnosis and treatment facilities as part of these policies should include county-level cancer registries to complement existing data. These registries can respond to the current gaps in Kenya’s cancer incidence and mortality data.

Cancer registries are required to provide data that will enable the country to support its cancer surveillance and plan for adequate resources to improve access to timely cancer diagnosis and treatment. The NCCS 2017–2022 indicates plans to improve and strengthen national cancer surveillance by establishing a National Cancer Registry using the Nairobi Cancer Registry, training personnel on health records and information systems, electronic data collection and quality assurance mechanisms. However, this requires establishing hospital-based cancer registries in each county to feed into the National Cancer Registry. To respond to these needs, the current cancer policies can be amended to designate of a portion of the budget allocated to cancer services to be applied in the establishment and management of county cancer registries as part of the national cancer registry. If implemented, county-level cancer registries could help address current gaps in Kenya’s cancer data and improve information for cancer policy formulation and implementation, resource planning and allocation. While a larger set of policy recommendations were identified in the study, only a limited set of these are presented based on their short-term viability. Other policy actions identified in this study can be undertaken in the long term.

### Limitations

This study’s policy review was limited by lack of studies on the impact and outcomes of Kenya’s cancer policies. Future studies can measure the current policy outcomes and their impact on improving access to cancer testing and treatment services in Kenya.

This policy analysis uses a limited subset of the population to provide opinions to inform recommendation. The generalisability of the interview responses is also limited by the small sample size (*n* = 14). Due to the significant barriers and delays of obtaining ethical approval to interview patients in Kenya as well as time and logistical constraints, we were unable to interview patients. Rather, our interviews were conducted with leaders of cancer patient support and advocacy groups to obtain cancer patients’ opinions. While excluding cancer patients might have reduced the strength of some of the issues articulated by the patient support and advocacy groups, two of the seven leaders of these groups are cancer survivors and were therefore able to offer a cancer patient’s perspective. Additionally, thematic saturation was attained after interviewing ten of the 14 participants.

## Conclusion

This study focused on documenting policy-modifiable factors and developing recommendations to improve policies affecting cancer testing and treatment services in Kenya. A review of Kenya’s cancer policies, results of a key informant survey and a stakeholder analysis were used to develop recommendations to improve cancer testing and treatment in Kenya. The study also identified progress made in developing Kenya’s cancer policies and guidelines and the government’s interest and commitment to improve access to cancer services in the country as well as gaps and opportunities for Kenya’s policy-makers and stakeholders to improve the formulation and implementation of cancer policies. Some of these opportunities include reviewing current cancer financing approaches, increasing stakeholder engagement and supporting county-level cancer surveillance. Future studies can explore the impact and outcomes of Kenya’s current cancer policies and guidelines.

## Data Availability

Data from the key informant survey is available upon request.

## Abbreviations

AMPATH: Academic Model Providing Access to Healthcare
KEMRI: Kenya Medical Research Institute
KENCO: Kenya Network of Cancer Organizations
MoH: Ministry of Health
MTRH: Moi Teaching and Referral Hospital
NCCS: National Cancer Control Strategy
NCI-K: National Cancer Institute Kenya
NCI-US: National Cancer Institute United States of America
NHIF: National Health Insurance Fund
UoN: University of Nairobi
